# Association Between Solid Cooking Fuels and Respiratory Disease Across Socio-Demographic Groups in India

**DOI:** 10.5696/2156-9614-9.23.190911

**Published:** 2019-08-22

**Authors:** Mohammad A. Faizan, Ramna Thakur

**Affiliations:** School of Humanities and Social Sciences, Indian Institute of Technology Mandi, Kamand Campus, Himachal Pradesh, India

**Keywords:** respiratory diseases, tuberculosis, bronchial asthma, solid fuels

## Abstract

**Background.:**

The use of solid fuels in household cooking contributes to indoor air pollution and is the cause of more than 4 million deaths around the world annually. Solid fuel use varies with the level of development and ranges from 0% in high-income countries to more than 80% in low- and middle-income countries. Three billion people (more than 40% of the global population) are still dependent on solid fuels like firewood, dung cakes, coal, wood and agricultural residues in these countries.

**Objectives.:**

The present study aims to analyze the association of certain respiratory diseases (tuberculosis (TB), acute upper respiratory infections (AURI), chronic obstructive pulmonary diseases (COPD), and bronchial asthma) with the use of solid fuels for cooking across sociodemographic groups in India.

**Methods.:**

The 71st round of the National Sample Survey, conducted in 2014, was used. In total, 54,985 inpatients who received medical treatment from any medical institution during the last 365 days preceding the survey and who reported various diseases, such as infections, cancers, blood diseases, cardiovascular diseases, and respiratory diseases were included in the analysis. Of these inpatients, 2513 participants who reported TB, AURI, COPD and bronchial asthma were considered the dependent variables in the study. The main variable was exposure to different types of fuels used as a primary source of energy for cooking. Multinomial logistic regression was used to explain associations.

**Results.:**

The results reveal a significant association between solid fuel use and respiratory diseases in India. Overall, more than 60% of the population uses firewood and cow dung as their primary source of energy for cooking and are at a higher risk of TB, COPD and bronchial asthma. In rural areas there is a high dependence on solid fuels (80.5%) and a higher risk of respiratory diseases compared to those residing in urban areas where people are less dependent on solid fuels (22%). Among different socio-demographic groups, the dependence on solid fuels is highest among Scheduled Tribes (87.42%), followed by Scheduled Castes (74.78%) and Other Backward Classes (OBCs) (a term used by the Indian government to categorize castes that face social or educational challenges) (64.47%). Scheduled Tribes have the highest risk of TB, followed by Scheduled Castes and OBCs, respectively.

**Conclusions.:**

Exposure to solid fuels for cooking increases the potential risk of TB, COPD and bronchial asthma. Access to clean and efficient fuels for cooking is essential to reduce the burden of respiratory disease. Measures are needed to increase the availability of clean fuels for households, especially among socially disadvantaged and marginalized groups, to reduce the burden of respiratory diseases in India.

**Competing Interests.:**

The authors declare no competing financial interests

## Introduction

Poverty is one of the greatest barriers to reducing global health inequalities, which are worsening in low- and middle-income countries.^1^ Dependence on inefficient fuels for household cooking and lighting is both a cause and a result of poverty.^2^ Rapid degradation of the environment and adverse impacts on human health caused by solid fuels has led to the search for solutions on a global scale.^3^ Use of solid fuels in household cooking is a major contributor to indoor air pollution, which causes more than 4 million deaths around the world annually.^4^,^5^ Solid fuel use varies with level of development, ranging from 0% in high-income countries to more than 80% in low- and middle-income countries such as China, India and sub-Saharan countries.^6^ Around the globe, three billion people (more than 40% of the global population) residing in low- and middle-income countries remain dependent on solid fuels such as firewood, dung cakes, coal, wood and agricultural residues.^5^,^7^ Most of these households use inefficient stoves filled with solid fuels for cooking and are poorly ventilated.^8^ Combustion of solid fuels generates many health-damaging pollutants, including respirable particulates, nitrogen oxides, benzene formaldehyde and carbon monoxide, exposure to which causes respiratory problems, such as acute lower respiratory infections, chronic obstructive pulmonary disease, lung cancer, pulmonary tuberculosis, and asthma.^3^,^6^ It also increases the risk of mortality and the burden of disease. Women are more affected by indoor air pollution as they spend more time in the kitchen and generally do all of the household cooking, which exposes them to toxic pollutants, smoke density and oil vapors, increasing the risk of respiratory infection.^9^ Infants and young children are also at high risk as they spend much of their time with their mothers in the kitchen. Furthermore, they have a higher rate of oxygen consumption per unit body weight than adults, and have narrower airways, which can result in inadequate ventilation and adds to the risk of respiratory infection.^3^,^10^

In India, more than 60% of the total population is dependent on solid fuels as their primary source of energy for cooking. India is a predominately rural country, and more than 70% of the population lives in rural areas, where more than 60% of the population is dependent on firewood and chips, and nearly 25% of the population uses crop residues and cow dung as a primary fuel for cooking.^11^ According to a report by the Government of India, 9% of medically reported deaths are due to diseases of the respiratory system and 30% of respiratory deaths are caused by pneumonia and asthma.^12^

In India, various studies have researched the association between respiratory diseases and the use of solid fuels for cooking and lighting. Many of these studies are based on a specific diseases, such as tuberculosis (TB), acute respiratory infections, and/or chronic obstructive pulmonary disorder (COPD).^13^–^16^ Most are regionspecific studies with a limited sample size, with the exception of the work conducted by Mishra *et al.* and Faizan and Thakur.^13^,^17^ Some of these studies are clinical studies and limited to a small sample size, although important in their own right. The present study aims to analyze the association of different respiratory diseases (TB, acute upper respiratory infections (AURI), COPD, and bronchial asthma) with the use of solid fuels for cooking across sociodemographic groups in India.

**Abbreviations**
*AURI*: Acute upper respiratory infections
*COPD*: Chronic obstructive pulmonary disorder
*LPG*: Liquefied petroleum gas
*OBC*: Other Backward Class
*RRR*: Relative risk ratio
*SC*: Scheduled Castes
*ST*: Scheduled Tribes
*TB*: Tuberculosis

## Methods

The present study is based on a National Sample Survey conducted in 2014, which included all the states and union territories of India. A multistage stratified sampling design was adopted, covering 65 932 households comprising 333 104 persons: 168 697 males, and 164 407 females. The details of the sampling design, survey tools and data collection methods are provided in the survey report.^18^ The survey collected information on 61 diseases and categorized them into 16 broader groups. Individuals who had received medical treatment as inpatients in a medical institution during the last 365 days preceding the survey were eligible the study. There were 55 026 persons who received medical treatment as inpatients (persons admitted to any medical institution for any medical treatment) and were diagnosed with various diseases. Only 54 985 individuals were analyzed in this study as 41 subjects had no cooking arrangements in their households and were dropped from the final analysis. Out of this group, 529 were diagnosed with TB, 520 with AURI, 345 with COPD and 1,160 with bronchial asthma, considered as dependent variables. Acute upper respiratory infections include cold, runny nose, sore throat with cough, and allergic colds. Chronic obstructive pulmonary disorder includes cough with or without fever, sputum, hemoptysis and marked breathlessness. Chronic obstructive pulmonary disorder excludes TB. The cooking fuels identified by the National Sample Survey and included in this study include coal, firewood and chips, liquefied petroleum gas (LPG), dung cake, charcoal, kerosene, electricity, others, or no cooking arrangements. In this study liquefied petroleum gas (LPG), electricity, and biogas are considered clean fuels and firewood, cow dung and coal products are considered as solid fuels.

Various socio-demographic variables were considered as covariates, as the association between energy use and chronic respiratory diseases can be confounded. Place of residence, caste, and gender were also taken into consideration. Table 1 provides the basic demographic profile of the population. Seventy percent (70%) and 30% of the population resides in rural and urban areas, respectively. Almost 64% of the population uses firewood, cow dung, coal products and kerosene as a primary source of energy for cooking. Eighty-one (81%) of the population are Hindu, followed by Muslim (14%), Christian (2.23%), or other religion (2.75%). Among different castes, 44% were classified as belonging to Other Backward Classes (OBC) (a term used by the Indian government to categorize castes that face social or educational challenges), followed by general (28%), Scheduled Castes (SCs) (19%), and Scheduled Tribes (STs) (9%).^19^–^21^

**Table 1:** Demographic Profile of the Study Population

**Variable**	**Sample population**	**Estimated population**	**% of total population**
**Source of energy for cooking**			
**Clean energy**	141 531	389 274 088	34.72
**Firewood**	160 713	605 823 491	54.04
**Cow dung**	18 860	81 273 916	7.25
**Coal products**	5499	18 730 922	1.67
**Kerosene**	3966	11 997 510	1.07
Others^*^	2373	12 873 740	1.15
**No cooking arrangement**	162	1 127 941	0.10
**Residence**			
**Rural**	189 573	784 954 357	70.02
**Urban**	143 531	336 147 252	29.98
**Caste**			
**General**	100 943	310 030 021	27.65
**Scheduled tribes**	43 142	103 799 864	9.26
**Scheduled castes**	55 454	211 205 534	18.84
**Other backward classes**	133 565	496 066 189	44.25
**Gender**			
**Male**	168 697	577 018 653	51.47
**Female**	164 407	544 082 955	48.53
**Religion**			
**Hindu**	251 924	909 519 177	81.13
**Muslim**	50 212	155 716 783	13.89
**Christian**	19 197	25 000 416	2.23
Other^**^	11 771	30 865 233	2.75
**Age**			
**0–14**	98 556	325 086 861	29.00
**15–29**	93 847	303 056 170	27.03
**30–44**	66 521	242 431 683	21.62
**45–59**	46 935	162 961 139	14.54
**60+**	27 245	87 565 756	7.81
**Education**			
**Illiterate**	103 001	353 501 025	31.53
**Literate through agency**	3511	11 248 856	1.00
**Up to middle school**	136 285	482 487 276	43.04
**Up to higher secondary**	64 268	202 229 843	18.04
**Graduate and above**	26 039	71 634 608	6.39
**Total**	**333 104**	**1 121 101 609**	**100.00**

^*^Others include other fuels apart from those mentioned

^**^ Other include Sikhs, Jains, Zoroastrians etc.

Stata 13 was used to conduct the statistical analysis. Multinomial logistic regression was used to explain the relationship between respiratory diseases and energy consumption. A meaningful interpretation of the results was done through relative risk ratio (RRR). The relative risk ratio is shown in Equation 1.

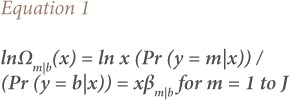



Predicted probabilities for J equation is shown in Equation 2.

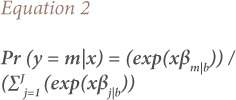
where, m represents respiratory diseases (TB, AURI, COPD and bronchial asthma), β are the coefficients for different variables and b is the base category (other diseases) or the comparison group.^22^ Since lnΩ_b|b_(x) = ln1 = 0, β_b|b_= 0 shows that the log odds of an outcome compared to itself is always 0, hence the effects of any independent variables must also be 0.

## Results

Table 2 shows the percent distribution of the primary source of energy for cooking across socio-demographic variables. It reveals that clean fuel in urban areas is used by two-thirds of the population, while nearly 80% of the population in rural areas is dependent on solid fuels. Socially disadvantaged and marginalized groups are highly dependent on solid fuels for cooking. Eighty-seven percent (87%) of those from STs and 75% of those from SCs are dependent on the solid fuels for cooking. Across religions, Muslims use the most solid fuels (64%) followed by Hindus (63%), Christians (56%) and others (52%).

**Table 2:** Percent Distribution of the Primary Source of Energy for Cooking Across Socio-Demographic Categories

**Variables**	**Clean energy**	**Firewood**	**Cow dung**	**Coal products**	**Kerosene^1^**	**Others**	**No cooking arrangement**
**Caste**							
**General**	52.38	37.02	5.18	2.09	1.59	1.60	0.15
**Scheduled Tribes**	11.76	84.58	1.67	1.17	0.43	0.37	0.03
**Scheduled Castes**	22.51	63.23	10.07	1.48	1.16	1.49	0.06
**Other Backward Class**	33.70	54.37	8.51	1.59	0.85	0.88	0.10
**Residence**							
**Urban**	74.59	17.51	1.89	2.60	2.88	0.22	0.30
**Rural**	17.65	69.68	9.55	1.27	0.30	1.55	0.01
**Religion**							
**Hindu**	34.63	54.58	7.37	1.39	0.98	0.96	0.09
**Muslim**	31.27	53.46	7.30	3.45	1.83	2.58	0.12
**Christian**	42.65	52.04	2.09	1.49	0.80	0.55	0.38
Other^*^	48.37	42.61	7.55	1.21	0.26	0.00	0.00

^*^Other includes Sikhs, Jains, Buddhists, Zoroastrians, and others.

The prevalence of respiratory disease varies across socio-demographic categories. Table 3 shows the rate of prevalence of respiratory diseases per hundred thousand people. The prevalence of TB was higher among kerosene users at 98 persons per hundred thousand, followed by individuals that use firewood and coal products at 47 and 46 persons per hundred thousand, respectively. The prevalence of bronchial asthma was highest among coal products, firewood, kerosene, and cow dung users at 186, 97, 85 and 80 persons per hundred thousand, respectively. Tuberculosis and COPD were more prevalent in rural areas at 46 and 30 persons per hundred thousand, respectively. Acute upper respiratory infections and bronchial asthma were more prevalent in the urban population at 52 and 121 per hundred thousand, respectively. Among various religious categories, TB was more prevalent among Muslims at 50 per hundred thousand, followed by Hindus at 44 persons per hundred thousand. Other diseases like AURI, COPD, and bronchial asthma were more prevalent among Christians at 70, 67 and 201 persons per hundred thousand, respectively, followed by Hindus and Muslims. Across castes, the prevalence of TB was higher among SCs and STs at 57 and 54 persons per hundred thousand, respectively. The prevalence of AURI was higher in the OBC and general category at 48 and 43 persons per hundred thousand, respectively. By gender, the prevalence of TB and COPD was higher among males than females at 54 and 33 persons affected per hundred thousand, respectively, while the prevalence of AURI and bronchial asthma was marginally higher in females than males. In the present study, the prevalence of respiratory disease increased with increasing age. Tuberculosis was more prevalent among older persons (age 60 and above), at 122 persons per hundred thousand affected, followed by 82 persons for those 45 to 59 years of age. The prevalence of AURI was also higher among older (> 60) persons, followed by the age group of 15–29 years of age at 92 and 75 persons per hundred thousand, respectively. The prevalence of bronchial asthma was very high among the elderly population compared to other respiratory diseases, with 627 persons affected per hundred thousand. The results also show that the prevalence of respiratory diseases among literate people is lower than illiterate individuals. Persons who are illiterate have a higher burden of respiratory disease than their educated counterparts across all respiratory diseases, including TB, AURI, COPD and bronchial asthma at 69, 72, 40 and 151 persons per hundred thousand population, respectively *(Table 3)*.

**Table 3:** Total Number of Estimated Cases and Prevalence Rate (per Hundred Thousand) of Respiratory Disease in Inpatients

**Variable**	**TB (per hundred thousand)**	**AURI (per hundred thousand)**	**COPD (per hundred thousand)**	**Bronchial asthma (per hundred thousand)**
**Source of energy for cooking**				
**Clean energy**	147 401 (38)	226 472 (58)	89 694 (23)	446 053 (115)
**Firewood**	286 717 (47)	220 276 (36)	178 859 (30)	585 932 (97)
**Cow dung**	31 727 (39)	17 046 (21)	32 037 (39)	64 836 (80)
**Coal products**	8709 (46)	2205 (12)	634 (3)	34 905 (186)
**Kerosene**	11 802 (98)	7133 (59)	3867 (32)	10 183 (85)
**Others**	12 794 (99)	5763 (45)	27 779 (216)	8102 (63)
**Residence**				
**Rural**	361 901 (46)	305 489 (39)	239 124 (30)	745 615 (95)
**Urban**	137 249 (41)	173 407 (52)	93 745 (28)	406 646 (121)
**Caste**				
**General**	122 772 (40)	133 442 (43)	98 997 (32)	380 760 (123)
**Scheduled Tribes**	55 721 (54)	20 684 (20)	22 735 (22)	49 675 (48)
**Scheduled Castes**	121 071 (57)	84 936 (40)	68 446 (32)	187 452 (89)
**Other Backward Class**	199 586 (40)	239 834 (48)	142 692 (29)	534 375 (108)
**Religion**				
**Hindu**	400 857 (44)	390 795 (43)	269 045 (30)	912 770 (100)
**Muslim**	78 153 (50)	67 440 (43)	43 717 (28)	144 512 (93)
**Christian**	8781 (35)	17 561 (70)	16 832 (67)	50 283 (201)
**Other**	11 359 (37)	3099 (10)	3275 (11)	44 697 (145)
**Gender**				
**Male**	314 075 (54)	245 067(42)	190 555 (33)	588 052 (102)
**Female**	185 075 (34)	233 829 (43)	142 315 (26)	564 210 (104)
**Age**				
**0–14**	117 978 (36)	60 189 (19)	45 047 (14)	69 668 (21)
**15–29**	63 789 (21)	226 241 (75)	74 443 (25)	167 070 (55)
**30–44**	77 386 (32)	47 618(20)	39 006 (16)	85 514 (35)
**45–59**	133 465 (82)	64 306 (39)	85 516 (52)	280 934 (172)
**60+**	106 531 (122)	80 541 (92)	88 858 (101)	549 075 (627)
**Education**				
**Illiterate**	242 211 (69)	253 678 (72)	142 280 (40)	534 839 (151)
**Literate through agency**	4365 (39)	2831 (25)	2158 (19)	16 493 (47)
**Up to middle school**	184 703 (38)	127 796 (26)	111 176 (23)	416 572 (86)
**Up to higher secondary**	58 694 (29)	55 463 (27)	69 368 (34)	147 944 (73)
**Graduate and above**	9176 (13)	391 27 (55)	7888 (11)	36 414 (51)
**Total (all India)**	565 676 (50)	503 855 (44)	35 078 (31)	1 286 200 (114)

Note: Values in parenthesis () is the prevalence rate of disease per hundred thousand

Table 4 shows the association between respiratory disease and type of energy used for cooking in households using multinomial logistic regression. In model 1, only the dependent variable, respiratory disease, and independent variable, the primary source of energy for cooking, have been included. In model 2, other variables, including place of residence, caste, and gender have been included along with the primary source of cooking fuel. Model 1 showed a significant association between TB and the use of firewood, cow dung and kerosene. The relative risk of TB was 75% higher among individuals who use firewood (RRR 1.75) as a primary source of energy for cooking, while the relative risk of TB increased nearly two-fold when cow dung (RRR 1.97) is used as a primary source of energy for cooking. In addition, the risk of TB was almost three times greater when kerosene is used as primary source of energy for cooking (RRR 2.84). The relative risk of COPD was 53% (RRR 1.53) and 56% (RRR 1.56) higher among firewood and cow dung users, respectively.

**Table 4:** Multinomial Logistic Regression of Socio-Demographic Categories and Respiratory Diseases

**Variables**	**Model 1**				**Model 2**			

	**TB**	**AURI**	**COPD**	**Bronchial asthma**	**TB**	**AURI**	**COPD**	**Bronchial asthma**
**Source of energy for cooking**								
**Clean energy**	1	1	1	1	1	1	1	1
**Firewood**	1.75^*^ (1.44–2.11)	0.94 (0.79–1.12)	1.53^*^ (1.22–1.93)	1.04 (0.92–1.18)	1.24^**^^*^ (0.99–1.55)	0.99 (0.80–1.23)	1 37^**^ (1.04–1.79)	1.17^**^ (1.01–1.35)
**Cow dung**	1.97^*^ (1.35–2.86)	0.69 (0.42–1.13)	1.56^***^ (0.96–2.54)	1.05 (0.79–1.39)	1.46^** *^ (0.98–2.17)	0.71 (0.42–1.18)	1.37(0.82–2.28)	1.13 (0.84–1.52)
**Coal products**	1.43 (0.70–2.93)	0.49 (0.18–1.31)	0.49 (0.12–2.00)	0.99 (0.61–1.62)	1.33 (0.65–2.73)	0.50 (0.18–1.33)	0.49 (0.12–1.97)	1.05 (0.64–1.71)
**Kerosene**	2.84^*^ (1.70–4.77)	1.21 (0.563–2.28)	1.72 (0.80–3.69)	0.75 (0.43–1.31)	2.76^*^ (1.64–4.64)	1.20 (0.63–2.26)	1.70 (0.79–3.67)	0.77 (0.44–1.34)
**Others**	2.31^*^ (1.02–5.24)	0.78 (0.25–2.45)	2.65^**^ (1.08–6.52)	1.13 (0.58–2.20)	1.81 (0.79–4.16)	0.84 (0.27–2.65)	2.49^**^ (1.00–6.22)	1.23 (0.63–2.42)
**Residence**								
**Urban**	-	-	-	-	1	1	1	1
**Rural**	-	-	-	-	1.51^*^ (1.22–1.88)	0.91 (0.74–1.12)	1.15 (0.89–1.50)	0.91 (0.79–1.05)
**Caste**								
**General**	-	-	-	-	1	1	1	1
**Scheduled Tribes**	-	-		-	2.10^*^ (1.58–2.78)	0.92 (0.66–1.28)	1.25 (0.86–1.82)	0.68^*^ (0.54–0.86)
**Scheduled Castes**	-	-	-	-	1.79^*^ (1.38–2.32)	1.12 (0.86–1.46)	(0.96–1.83)	0.82^**^ (0.69–0.99)
**Other Backward Class**	-	-	-	-	1.28^**^ (1.01–1.62)	1.08 (0.88–1.33)	1.24 (0.95–1.63)	0.91 (0.79–1.04)
**Gender**								
**Male**	-	-	-	-	1	1	1	1
**Female**	-	-	-	-	0.34^*^ (0.28–0.41)	0.48^*^ (0.40–0.57)	0.40^*^ (0.32–0.50)	0.45^*^ (0.40–0.51)

^*^significant at 1%,

^**^ significant at 5%,

^***^significant at 10%, values in parenthesis () is confidence interval.

Model 2 also showed a significant association between TB, COPD and use of firewood, cow dung and kerosene. The relative risk of TB declined to 24% in model 2 for firewood users (RR 1.24) and 46% for cow dung users (RR 1.46) compared to model 1. Model 2 also showed an association of respiratory diseases between place of residence, caste, and gender apart from cooking fuel. Among the rural population, the relative risk of TB was higher (RR 1.51) compared to their urban counterparts. Among different social categories, the relative risk of TB was highest among those from SCs (RR 2.10), followed by STs (RR 1.79) and OBCs (RR 1.28). However, the risk of bronchial asthma among SCs (RR 0.68) and STs (RR 0.82) was significantly lower than persons in the general category. The relative risk for gender of any respiratory disease was lower among females than males.

## Discussion

The present study found that the use of solid fuels had a significant association with respiratory disease. Use of firewood, cow dung and kerosene as the primary sources of energy for cooking showed a significant association with TB, COPD and bronchial asthma. Using firewood and cow dung as fuels releases pollutants such as carbon monoxide, carbon dioxide, sulfur dioxide, nitrogen dioxide, volatile organic compounds and/or hydrocarbons.^3^,^6^,^8^ Particulate matter PM_10_ and PM_2.5_ are also released, which is a primary cause of respiratory diseases.^6^,^23^–^39^ The use of kerosene as a fuel for cooking is widespread in developing countries.^30^ Kerosene is burned mainly on stoves, such as wick stoves (low efficiency) or pressure stoves (more efficient). When low efficiency stoves are used to burn kerosene, it emits health damaging pollutants that can lead to lung function impairment, asthma, infectious diseases, cataract and is toxic to children.^30^–^32^

Since independence, India has seen significant progress in many development indicators, such as reductions in poverty, death rate and infant mortality, increases in gross domestic product and life expectancy, and improvement in the human development index, among others. However, access to clean energy for cooking is still lacking among various sections of society, especially socially disadvantaged and marginalized groups. These groups are economically vulnerable and are more dependent on solid fuels for cooking, putting them at higher risk of respiratory diseases. Most of these individuals live in areas which are isolated, remote, or ghettoized and face barriers to accessing clean fuels.^33^ Individuals belonging to SCs are mostly segregated in areas generally outside the perimeters of villages in rural areas, poorer neighborhoods and in slums in urban areas. People belonging to STs often live in remote locations, particularly in forested areas, and have minimal access to markets, which deprives them of basic amenities such as clean fuels, safe drinking water and sanitation facilities.^34^ Furthermore, Muslims generally live in urban areas, within city boundaries.^35^ Marginalization and lack of basic facilities, particularly access to clean fuels for cooking, have a significant effect on the outcome of respiratory diseases.^36^,^37^ However, in the present study, the risk of bronchial asthma among persons belonging to SCs and STs was lower than individuals belonging to the general category. People belonging to SCs and STs generally live in rural areas. Individuals from the general category and OBCs tend to live in urban areas where exposures to dust mites, emissions from vehicles, and indoor biological contaminants occur more frequently. The results of the present study are consistent with other studies that show the occurrence of COPD related symptoms are more prevalent in urban areas.^38^–^40^

In India, solid fuel is the primary source of energy for cooking in rural households and a frequent fuel source in urban homes as well. Cooking areas play an important role in Indian households, where women do most of the household cooking, as well as maintaining the kitchen and raising children. Hence, women are more greatly exposed to the harmful effects of the solid fuels compared to men. The concentration of particulate matter and percentage of toxins is highest in the kitchen, which has been associated with various respiratory diseases among women and children.^9^,^28^,^41^ However, the present study suggests that the relative risk of respiratory diseases such as TB, AURI, COPD and bronchial asthma is lower among females than males. It may be that males are more vocal and expressive regarding their health issues than females due to social and economic factors. Cigarette smoking is a major cause of respiratory disease and males tend to have a higher rate of smoking than females, which may confound the results.^42^–^45^ Another reason could be that males are more exposed to smoke from passive cooking effects and are exposed to dust while working on farms and construction projects.^9^,^46^ Physiological differences, such as smaller diameter airway, lung volume, maximum expiratory flow and diffusion surface in women compared to men could also affect the study results.^47^

While many believe the poor cannot afford to use clean fuels for cooking and lighting, the reality is a lack of access to clean fuels.^48^ Inaccessibility of clean fuels for cooking is a root cause of poverty, poor health, gender inequality, environmental degradation, and air pollution and contributes to climate change.^49^ Exposure to smoke from solid fuel use is a major environmental risk factor, contributing to 3.3% of all deaths worldwide.^50^ Access to clean and efficient fuels for cooking should be prioritized for every section of society, with a particular focus on socially disadvantaged, marginalized and rural populations. This will undoubtedly help to reduce the burden of respiratory diseases. The number of households without clean cooking fuel in India is more than double the number of those without access to electricity, which not only impacts the health of the households, but their finances as well.^51^ In addition to the health and monetary costs, the collection of firewood and chips consumes time and energy which could be used towards childcare, education, or leisure, etc.^52^

The first attempt to collect data on health in India began in the 1950's in the 7^th^ round of the National Sample Survey. The health survey was also conducted in three subsequent rounds, the 11^th^ to 13^th^. However, the first full scale survey on morbidity was conducted on the 28^th^ round. Other national health surveys began in the 1990's with the National Family Health Survey in 1992 and the District Level Health Survey in 1998. These surveys mainly focused on maternal and child health, which were prominent health issues in India at the time.

However, over the years the burden has significantly shifted towards non-communicable diseases. Over the last few years there has been an increase in non-communicable diseases and injuries, which account for 55% and 12% of the total burden of disease, respectively, however this change was not evident in the latest health surveys in India.^53^–^55^ Reliable information on respiratory diseases in India is lacking and there is a need to collect more information, including indoor air quality. The various health surveys should be consolidated into a single survey, where information on various diseases and associated factors can be collected within a 5-year gap. This will provide better insights on health statistics and be useful in making suitable policy interventions.

There is also a need to create public awareness regarding the negative health consequences of the use of solid fuels. Technologies should be developed to provide safe and clean energy for cooking such as biomass briquettes and biogas. India should ensure the strengthening of the financial ecosystem to enable affordable access to sustained use of clean fuels. Local communities should be involved in technology development and application, especially in building biogas plants from non-gobar (non cow dung) products.

In 2016, the Government of India initiated the Pradhan Mantri Ujjwala Yojana to provide LPG connections to 50 million households by 2019 in order to prevent deaths and illness from hazardous pollution and to ensure women's empowerment, particularly in rural India.^56^ As of early January 2019, 60 million connections were provided under the scheme.^57^ However, the success of this scheme in realizing health benefits does not depend solely on the distribution of LPG connections, but also on transitioning households away from using traditional biomass fuels to the use of modern fuels.^58^,^59^ The major concern of consumers has been the initial and refill cost of LPG cylinders, which has hindered a complete switch to modern fuels.^60^ There is a need to reduce the cost of clean energy by putting greater emphasis on the research and development of alternative clean sources of energy for cooking.

### Future research

Despite the contribution of research on the understanding of disease in general, information on actual exposures and burden of disease due to indoor air pollution is lacking. Further studies are needed to collect information on indoor air pollution in order to obtain good quality data to help shape better policy interventions.

### Limitations

The National Sample Survey has been conducting national surveys on health since 1972 and has been an invaluable source of health information in India. However, health information in India has not kept up with epidemiological transitions, leading to inadequate information. In 2016, the burden of non-communicable diseases accounted for 55% of the total disease burden in India, but adequate information has not been collected on these diseases. Although the 71^st^ round of the National Sample Survey included information on respiratory diseases like TB, AURI, COPD, and bronchial asthma, it still lacks information on various important confounding variables such as lung development, physical activity, active smoking, time spent in the kitchen, level of pollution in households, and concentration of particulate matter. These factors are also important for understanding the effect of solid fuel use on respiratory diseases. There is a need for a holistic health data survey in India which will provide risk factors of major diseases and can help researchers explore the association between different fuels used for cooking and respiratory diseases. This research will surely play an important role in framing health policies and targeting health priorities and interventions.

## Conclusions

Many studies have found an association between the use of solid fuels as primary source of energy for cooking and respiratory diseases, but empirical evidence has been limited. The present study takes into consideration these issues and demonstrates the association between use of solid fuels as a primary source of energy for cooking and respiratory diseases using nationally representative data. Exposure to solid fuels for cooking increases the potential risk of TB, COPD and bronchial asthma among socially and economically marginalized groups. The present study found that the respiratory health of females is not more greatly affected by the use of solid fuels. However, there is a need for dedicated gender-focused research, which will provide a better understanding of the role of solid fuels and its association with respiratory diseases in females. Melinda Gates once said “we cannot close the gender gap without first closing the data gap” and hence gender-focused data on energy consumption in households should to be a priority.^61^ There is need to further strengthen the Pradhan Mantri Ujjwala Yojana to make it more accessible and affordable for poor households in rural areas by incorporating more departments and agencies. In addition, emphasis is needed on the promotion of alternate sources of cleaner fuels for cooking apart from LPG.
